# Surveillance of deaths caused by arboviruses in Brazil: from dengue to chikungunya

**DOI:** 10.1590/0074-02760160537

**Published:** 2017-08

**Authors:** Luciano Pamplona de Góes Cavalcanti, André Ricardo Ribas Freitas, Patrícia Brasil, Rivaldo Venâncio da Cunha

**Affiliations:** 1 Universidade Federal do Ceará, Departamento de Saúde Comunitária, Fortaleza, CE, Brasil; 2 Faculdade São Leopoldo Mandic, Campinas, SP, Brasil; 3 Secretaria Municipal de Saúde, Departamento de Vigilância em Saúde, Campinas, SP, Brasil; 4 Fundação Oswaldo Cruz-Fiocruz, Instituto Nacional de Infectologia Evandro Chagas, Rio de Janeiro, RJ, Brasil; 5 Fundação Oswaldo Cruz-Fiocruz, Campo Grande, MS, Brasil

**Keywords:** surveillance, death, arboviruses, dengue, chikungunya

## Abstract

Did death occur DUE TO dengue, or in a patient WITH dengue virus infection? It seems a matter of semantics, but in fact, it underscores how challenging it is to distinguish whether the disease contributed to death, or was itself the underlying cause of death. Can a death be attributed to chikungunya virus, when some deaths occur after the acute phase? Did the virus decompensate the underlying diseases, leading to death? Did prolonged hospitalisation lead to infection, resulting in the patient’s progression to death? Were there iatrogenic complications during patient care? The dengue question, for which there has not yet been a definitive response, resurfaces prominently under the chikungunya surveillance scenario. We are facing an epidemic of a disease that seems to be more lethal than previously thought. The major challenge ahead is to investigate deaths suspected of occurring due to arbovirus infections and to understand the role of each infection in the unfavourable outcome.

The circulation of dengue virus (DENV) in Brazil was only definitively proven in 1982, when the DENV-1 and DENV-4 viruses were isolated in Boa Vista, the capital of the former federal territory of Roraima (Osanai et al. 1983). However, there have been reports of dengue outbreaks in the nineteenth and twentieth centuries (Rego 1872, Reis 1896, Mariano 1917, Pedro 1923). The disease was viewed as one with high epidemic potential, with an expected lethality in severe forms of less than 1% (Osanai 1984). The first major epidemic of dengue occurred in 1986/87 (Teixeira et al. 1999); the first severe cases were reported since 1990 with the introduction of the second serotype DENV-2 (Schatzmayr 2000, Silva Jr et al. 2002, Cavalcanti et al. 2010). Since then, the challenge faced in association with training of professionals in the management of patients and organisation of care for the treatment of severe cases has become a serious concern.

Despite all of the government’s efforts, the lethality rate due to dengue virus in Brazil has remained higher than the optimal rate recommended by the World Health Organization (Teixeira et al. 2013). It is important to emphasise that all dengue epidemics are predictable, months before they are established, and that the deaths resulting from these epidemics are almost entirely preventable. A network of health services is required for this, and should be carefully organised by advanced preparations in order to reduce mortalities (Cunha & Martinez 2015).

Even after the significant advances over more than 30 years of dengue surveillance in Brazil, it has only recently been recognised that there are many deaths that are as yet not being detected by the health services. In Brazilian cities between 2011 and 2012, with organised, structured autopsy services and the use of surveillance and laboratory teams, the lethality rate due to dengue has tripled, suggesting that in many places the number of dengue-related deaths is underestimated (Braga 2014, Cavalcanti et al. 2016).

Although considerable advances have been made in the field, and knowledge regarding dengue has been revealed, epidemiological surveillance and death investigation committees have faced challenges in determining whether a death occurred DUE TO dengue virus or in a patient WITH dengue virus infection. This question reflects the difficulty in establishing whether the disease contributed to death, or was in fact the underlying cause of death. Importantly, this is an acute infectious disease in which most of the documented deaths usually occur prior to day 10 of illness (Campos et al. 2015, Cavalcanti et al. 2016).

Why is this question relevant now? Because we are in the process of commencing the organisation and systematisation of information and scientific evidence regarding the history of chikungunya fever in Brazil (Vasconcelos 2014, Donalísio & Freitas 2015, Honório et al. 2015).

Following the isolation of chikungunya virus (CHIKV) in 1952 in Tanzania, the virus has been identified in Southeast Asia and India, establishing an urban transmission cycle that continues today. The second emergence of CHIKV occurred in Kenya in 2004, with the virus spreading over many islands of the Indian Ocean in the following years and reaching India and Southeast Asia. Between 2005 and 2006, an epidemic hit the islands of Reunion. At the end of 2013, the Pan American Health Organization issued an epidemiological alert due to the detection of the first local cases of chikungunya in the Americas. In August 2015, autochthonous transmission had been detected in 33 countries and territories of the Americas, and Latin America reported almost one million cases (PAHO 2011, Yakob & Clements 2013).

In Brazil, autochthonous transmission of CHIKV was simultaneously detected in September 2014 in Feira de Santana (Bahia) and Oiapoque (Amapá). During 2014, there were 2,772 confirmed cases of CHIKV in six Federative Units. In 2015, 38,332 probable cases were reported in the country, distributed over 696 municipalities (MS/SVS 2014, 2016, Teixeira et al. 2015).

Before the outbreak on Reunion Island, this disease was not associated with high fatality rates (Economopoulou et al. 2009). In recent years, however, many studies have challenged the conventional view of the non-lethal nature of CHIKV (Economopoulou et al. 2009, de la Hosz et al. 2015). The severe form of CHIKV infection can be associated with multiple organ failure, hepatitis, meningitis, nephritis, encephalitis, bullous dermatitis, myocarditis, and cardiac arrhythmias. While severe or atypical manifestations of CHIKV infection are uncommon, the overall fatality rate of these complications appears to be high (Couderc & Lecuit 2015).

It was during the outbreak of chikungunya that affected Reunion Island in 2005-2006 that the severity of neonatal forms of infection, acquired by transmission from mother to child during childbirth, was observed. When the mother is viraemic at the time of delivery, the rate of mother-to-child transmission is about 50%. All neonates contaminated during labour and delivery present with symptomatic disease and the rate of severe forms is approximately 50%, primarily due to damage to the central nervous system, which often results in permanent, severe outcomes such as seizures and cerebral palsy (Gerardin et al. 2008). While CHIKV infection is not recognised among neonatal sepsis cases, the burden of neonatal complications due to this alphavirus may also be underestimated.

From January to August 2016, about 220,000 cases of chikungunya have been reported in Brazil, indicating a troubling scenario with respect to morbidity and mortality. The current epidemic has the potential to be explosive, reaching great magnitudes because of the large population of susceptible individuals and the wide-reaching spread of its main vector. This is likely to result in many suspicious deaths, mainly in the northeast of the country where more than 90 deaths were confirmed from January to August 2016 (MS/SVS 2014, 2016). Brazil has been slow to confirm cases, with difficulties associated with the identification of deaths through information systems, despite mandatory reporting of CHIKV infection within 24 h. Therefore, these data on CHIKV deaths in Brazil are likely to be still underestimated, as was the case on Reunion Island (2005/2006), where less than one-third of deaths were reported (Josseran et al. 2006).

The Ministry of Health of Brazil has appropriately adapted the dengue death investigation protocols by changing them to arbovirus death investigation protocols, owing to the triple occurrence of dengue, chikungunya, and zika (Carvalho & Cavalcanti 2016, Coelho et al. 2016).

As a diagnosis of dengue can be confirmed serologically after 7-10 days, many deaths during the first week are “probable cases”; this is a limitation in the investigation of deaths. Distinguishing underlying disease or causes of death is impossible during surveillance for most diseases, and useless from the perspective of a transmissible disease. However, it is necessary to know the fatality rates in order to direct actions of surveillance and control.

The current challenge is to investigate and classify deaths caused by chikungunya, considering that patients can die not only in the acute phase (up to 21 days), but also in the subsequent post-acute phase (22 days to three months post-infection) or even in the chronic phase (> 3 months), due to complications triggered by the virus itself. The cause of death cannot be confirmed by direct methods [reverse transcriptase polymerase chain reaction (RT-PCR) or virus isolation], especially in the post-acute or chronic phases (MS/SVS 2016).

The question remains how one can assign a particular cause of death to chikungunya. Did chikungunya infection decompensate underlying diseases, which led to death? Did the disease progress unsatisfactorily owing to the presence of another underlying disease? Did the disease engender the need for a prolonged hospitalisation, leading to nosocomial infection, and subsequent progression to death? Was the therapeutic management inadequate? Was there an iatrogenic complication during patient care? What is the role of neurological complications in the causation of death due to CHIKV? Whether chikungunya is the basic or underlying cause of deaths, the associated co-morbidities, secondary infections and inappropriate use of non-steroidal anti-inflammatory drugs appear to contribute to the fatal outcome.

Thus, the question, for which there has not yet been a definitive response in relation to dengue virus infections, resurfaces prominently with regard to the chikungunya surveillance scenario in Brazil. In the case of a patient that has died, did chikungunya have a secondary role, or was CHIKV infection the underlying cause of death? Irrespective of whether a patient’s death was solely due to CHIKV infection, due to unrelated causes, or due to an interaction between CHIKV infection and other causes, we should be concerned about the epidemic of a disease that we are facing that may be more lethal than previously thought. We have a major challenge ahead to appropriately capture and investigate deaths suspected of being caused by arbovirus infections and to understand the role of each virus in the unfavourable outcome. A comparable database is important to help us understand this disease and its pathophysiology, as well as the impact of the association between arboviruses and underlying diseases.

Moreover, specific protocols need to be developed regarding the medical attention given to patients with a suspected and/or clinical diagnosis of chikungunya associated with co-morbidities, with the aim of enhancing clinical management in order to reduce deaths.
